# LncRNA *SNHG16* promotes colorectal cancer proliferation by regulating ABCB1 expression through sponging miR-214-3p

**DOI:** 10.7555/JBR.36.20220049

**Published:** 2022-06-28

**Authors:** Pei Tan, Mu Xu, Junjie Nie, Jian Qin, Xiangxiang Liu, Huiling Sun, Shukui Wang, Yuqin Pan

**Affiliations:** 1 General Clinical Research Center, Nanjing First Hospital, Nanjing Medical University, Nanjing, Jiangsu 210006, China; 2 Department of Laboratory Medicine, Nanjing First Hospital, Nanjing Medical University, Nanjing, Jiangsu 210006, China; 3 School of Medicine, Southeast University, Nanjing, Jiangsu 210009, China; 4 Jiangsu Collaborative Innovation Center on Cancer Personalized Medicine, Nanjing Medical University, Nanjing, Jiangsu 211100, China

**Keywords:** *SNHG16*, ATP-binding cassette subfamily B member 1, microRNA, colorectal cancer, ceRNA

## Abstract

Mounting evidence indicates that long non-coding RNAs (lncRNAs) have critical roles in colorectal cancer (CRC) progression, providing many potential diagnostic biomarkers, prognostic biomarkers, and treatment targets. Here, we sought to investigate the role and underlying regulatory mechanism of lncRNA small nucleolar RNA host gene 16 (*SNHG16*) in CRC. The expressions of *SNHG16* in CRC were identified by RNA-sequencing and quantitative reverse transcription PCR. The functions of *SNHG16* were explored by a series of *in vitro* and *in vivo* assays (colony formation assay, flow cytometry assay, and xenograft model). Bioinformatics analysis, RNA fluorescence*in situ* hybridization and luciferase reporter assay were used to investigate the regulatory mechanism of effects of *SNHG16*. *SNHG16* was found to be significantly elevated in human CRC tissues and cell lines. Functional studies suggested that *SNHG16* promoted CRC cell growth both *in vitro* and *in vivo*. Mechanistically, we identified that *SNHG16* is expressed predominantly in the cytoplasm. *SNHG16* could interact with miR-214-3p and up-regulated its target ABCB1. This study indicated that *SNHG16* plays an oncogenic role in CRC, suggesting it could be a novel biomarker and therapeutic target in CRC.

## Introduction

Colorectal cancer (CRC) is an invasive primary intestinal malignant disease with the third highest incidence and mortality among all cancers in the world^[1]^. Due to the lack of early symptoms or effective screening methods, many CRC patients are diagnosed at advanced stages^[2]^. In addition, recurrence, metastasis and drug resistance are closely related to the poor prognosis of CRC patients^[3]^. Thus, a detailed understanding of the molecular mechanism of CRC occurrence and progression is urgently needed to improve early diagnosis and treatment techniques.

Long non-coding RNAs (lncRNAs) are RNA molecules greater than 200 nucleotides in length^[4]^. Accumulating studies have shown that lncRNAs regulate gene expression in a variety of ways, such as epigenetic regulation, and interaction with RNA and DNA^[5–6]^. Increasing evidence has shown that lncRNAs are closely related to the tumorigenesis and development of CRC, indicating that lncRNAs could be potential targets for CRC treatment^[7–8]^.

Small nucleolar RNA host gene 16 (*SNHG16*) is located on 17q25.1 and was recently identified as a cancer-associated lncRNA. It has been demonstrated to be associated with numerous cancers, such as bladder cancer, osteosarcoma and hepatocellular carcinoma^[9–12]^. Li *et al* reported that *SNHG16* expression was increased in CRC and was correlated with the poor prognosis of CRC patients^[13]^. However, the functions of *SNHG16* in CRC remain to be elucidated.

ATP-binding cassette subfamily B member 1 (ABCB1) belongs to the superfamily of human adenosine triphosphate (ATP)-binding cassette (ABC) transporters that encode transporter and channel proteins that function as efflux pumps. Wang *et al* demonstrated that abnormal expression of ABCB1 promoted the proliferation and metastasis of hepatocellular carcinoma cells by regulating nuclear factor-κB expression^[14]^. Yan *et al* showed that ABCB1 plays a vital role in promoting angiogenesis and metastasis in xenograft tumor models with CRC liver metastasis^[15]^.

In this study, we aimed to identify and characterize lncRNAs functionally impacting CRC. By analyzing datasets downloaded from The Cancer Genome Atlas (TCGA) and Gene Expression Omnibus (GEO), we found that lncRNA *SNHG16* was significantly overexpressed in CRC tissues and cell lines. *SNHG16* silencing inhibited CRC cells growth *in vivo* and *vitro*, and we proved that *SNHG16* acted as a ceRNA in regulating ABCB1 through competitively binding to miR-214-3p. Our findings suggested that *SNHG16* could be a potential biomarker and treatment target in CRC.

## Materials and methods

### Cell culture

Human CRC cell lines (HCT116, HT29, SW480, SW620, and Caco2), human normal colorectal epithelial cell line NCM460 and human renal epithelial cells (293T) were obtained from the American Type Culture Collection (USA). SW620 and SW480 cells were cultured in Leibovitz's L15 medium (L15) (Cat. No. GNM-41300, Genom, China) with 10% fetal bovine serum (FBS) (Gibco, Austria), and all other cells (HCT116, HT29, Caco2, and 293T) were grown in Dulbecco's modified Eagle's medium (DMEM) (Cat. No. GNM-12800, Genom) with 10% FBS. NCM460 cells were maintained in RPMI 1640 medium (Cat. No. GNM-31800, Genom) with 10% FBS. All cells were incubated in a humidified atmosphere at 37 °C with 5% CO_2_.

### Patient samples

Twenty paired primary tumor tissues (T) and corresponding nontumor tissues (N) were collected from patients who underwent primary surgical resection at Nanjing First Hospital affiliated with Nanjing Medical University from January 2018 to June 2020. None of these patients received radiotherapy or chemotherapy before surgery. Corresponding nontumor tissues were collected >2 cm from the tumor margin by histopathologic review. After surgical resection, tissues were immediately snap frozen and stored in liquid nitrogen. This study protocol was approved by the Ethics Committee on Human Research of the Nanjing First Hospital affiliated with Nanjing Medical University. The ethical file number of the study was (2017)-155. Moreover, informed consent was obtained from all patients.

### Vector construction and cell transfection

Small hairpin RNAs (shRNAs) targeting *SNHG16*, the *SNHG16*-expressing vectors, siRNAs, microRNA mimics and inhibitors were purchased from GeneChem (China). Cells were plated in six-well plates and cultured to 30% to 50% confluence in a complete medium before being infected with lentiviral vectors at a multiplicity of infection of 10 following the manufacturer's protocol. To select stable cell lines, cells were maintained in a medium with 2 μg/mL puromycin for 14 consecutive days. siNC and siRNAs were transfected using Lipofectamine 2000 (Invitrogen, USA) following the manufacturer's guidelines. All siRNA and shRNA sequences are listed in ***Supplementary Table 1*** (available online).

### RNA extraction and quantitative reverse transcription PCR

Total RNA was extracted from cells and tissues by using TRIzol reagent (Invitrogen) in accordance with the manufacturer's protocol. LncRNA and mRNA detection was performed using a SYBR Green PCR Kit (Takara, Japan) with an ABI 7500 System (Applied Biosystems, USA). GAPDH was used as the reference gene. MicroRNA detection was performed using a Hairpin-iT microRNA and U6 snRNA Normalization RT-PCR Quantitation Kit (GenePharma, China). For *SNHG16* copy number detection, total RNA was reverse transcribed into complementary DNA (cDNA) using a PrimeScript RT reagent kit (Takara), and quantitative reverse transcription PCR (qRT-PCR) was carried out with TB Green Premix Ex Taq Ⅱ (Tli RNaseH Plus) (TaKaRa) on an ABI 7500 real-time PCR system. Fold changes were analyzed using the 2^–ΔΔCT^ method and normalized to the *GAPDH* or *U6* gene. Primers sequences were as follows: *SNHG16* forward, 5′-GATCCCATCTGGCATCGCT-3′ and reverse 5′-CCTCTAGTAGCCACGGTGTG-3′; U6 forward, 5′-CTCGCTTCGGCAGCACA-3′ and reverse 5′-AACGCTTCACGAATTTGCGT-3′; *GAPDH* forward, 5′-GGGAGCCAAAAGGGTCATCA-3′ and reverse 5′-TGATGGCATGGACTGTGGTC-3′; *ABCB1* forward, 5′-CGAGGTCGGAATGGATCTTGA-3′ and reverse 5′-CCAAAGTTCCCACCACCATATAC-3′.

### Western blotting analysis

Cells were harvested with RIPA lysis buffer supplemented with PMSF, protein inhibitors and phosphatase inhibitors (KeyGEN, China). Then, the cell protein lysates were quantified, separated by SDS-polyacrylamide gel electrophoresis, and transferred to PVDF membranes (Millipore, USA). Membranes were blocked with 5% nonfat milk and then probed with specific antibodies: PARP (Cat. No. 13371-1-AP, Proteintech, USA; 1:2000 dilution), Bax (Cat. No. 50599-2-Ig, Proteintech; 1:5000 dilution), GAPDH (Cat. No. 60004-1-Ig, Proteintech; 1:2000 dilution), peroxidase conjugated goat anti-mouse IgG (Biosharp, China; 1:5000 dilution), peroxidase conjugated goat anti-rabbit IgG (Biosharp; 1:5000 dilution). Protein expression was detected using the bioimaging system ECL Plus (Millipore) after incubation with secondary antibodies.

### Colony formation assay

After transfection, cells were initially plated in 6-well plates (500 cells per well) and incubated at 37 °C for 14 days. Next, the colonies were fixed in 4% paraformaldehyde following treatment with 5% crystal violet (Beyotime, China). The ability of the cells to proliferate was determined by the number of colonies stained. The number of colonies in five fields of view was counted under an Olympus FSX100 microscope (Olympus, Japan).

### Apoptosis Assay

Apoptosis assays were performed using the Annexin V-FITC apoptosis detection kit (KeyGEN) following the instructions. Briefly, after transfections, cells were collected and washed with cold PBS at 4 °C twice, followed by treatment with Annexin V-FITC and propidium iodide (PI) in the dark for 15 minutes at room temperature. A FACSCalibur flow cytometer (BD Biosciences, USA) was used to detect apoptosis.

### RNA fluorescence *in situ* hybridization

Fluorescence *in situ* hybridization (FISH) assays were performed using a Fluorescent In Situ Hybridization Kit (RiboBio, China) in accordance with the manufacturer's guidelines. Cy3-labeled SNHG16 probes were designed and synthesized by RiboBio. Briefly, cells were washed twice with PBS before being fixed in 4% formaldehyde for 15 minutes. Then, the fixed cells were permeabilized with 0.5% Triton X-100 at 4 °C for 30 minutes and prehybridized at 37 °C for 30 minutes in prehybridization solution. The probes and cells were then incubated in the hybridization solution at 37 °C overnight in the dark. The cells were scanned and imaged after being treated with DAPI working solution.

### Subcellular fractionation

The nuclear and cytosolic fractions were separated using a PARIS Kit (Invitrogen) following the manufacturer's protocol.

### *In vivo* experiments

For the subcutaneous tumor xenograft model, five- to six-week-old BALB/c nude male mice were kept in a specific pathogen-free environment. All animal experimental procedures were carried out in accordance with the protocol authorized by the Animal Care Committee of Nanjing Medical College. Stably transfected HCT-116 cells or negative control (5×10^6^ cells/0.2 mL PBS) were inoculated into the same mice. Weekly tumor size was measured with calipers, and the tumor volume calculation formula was as follows: (L×W^2^)/2. After one month, the mice were euthanized, and the volumes and weights of tumor were recorded.

### Immunohistochemistry analysis

CRC tissues and normal tissues were collected and embedded in paraffin. The section thickness was 4 μmol/L. After being treated with fractionated ethanol and distilled water, sections were treated with methanol and 3% H_2_O_2_ for 30 minutes. Sections were washed three times with PBS and incubated with 10% goat serum for 30 minutes to prevent nonspecific antibody binding. After washing, sections were incubated with anti-ABCB1 (Cat. No. ab170904, Abcam, USA) antibodies at 4 °C overnight, followed by incubation with secondary antibodies. Following the manufacturer's protocol, the sections were stained with diaminobenzidine, sealed and visualized under an Olympus FSX100 microscope.

### Dual-luciferase reporter assay

To verify the direct interactions between *SNHG16* and miR-214-3p, the *SNHG16* 3′ untranslated region (3′ UTR) fragment containing a wild type (Wt) or mutant (Mut) binding site of miR-214-3p was amplified by PCR and inserted into the PmirGLO vector (GeneCreat, China). 293T cells were cotransfected with sh-miR-214-3p/sh-NC reagent (Invitrogen). After transfection for 48 hours, cells were collected. Results were measured by Dual-luciferase Reporter analysis system (Promega, USA) with normalization to Renilla luciferase.

### Bioinformatics analysis

The TCGA data used in our study were pre-analysis RNA-seq (level 3) data downloaded from the GDC database (41 normal tissues and 478 CRC tissues). We also used two gene array data of CRC downloaded from the GEO. The 'Tsukamoto's Colorectal' cohort (GSE21510), which has 44 samples (normal is 25 and tumor is 19), and the 'Okazaki's Colorectal' (GSE22598), which has 34 samples (normal is 17 and tumor is 17).

### Statistical analysis

Representative results were obtained through at least three independent experiments. The data are expressed as mean±SD. Statistical analysis was performed by SPSS 19.0 software (IBM, USA), and images were acquired with GraphPad Prism 7 software (GraphPad Software, USA). The significance of the differences between the groups was compared using Student's paired or unpaired *t*-tests or one-way ANOVA or chi-square test, respectively. *P*<0.05 was considered statistically significant.

## Results

### *SNHG16* expression was upregulated in colorectal cancer

**Figure 1:**
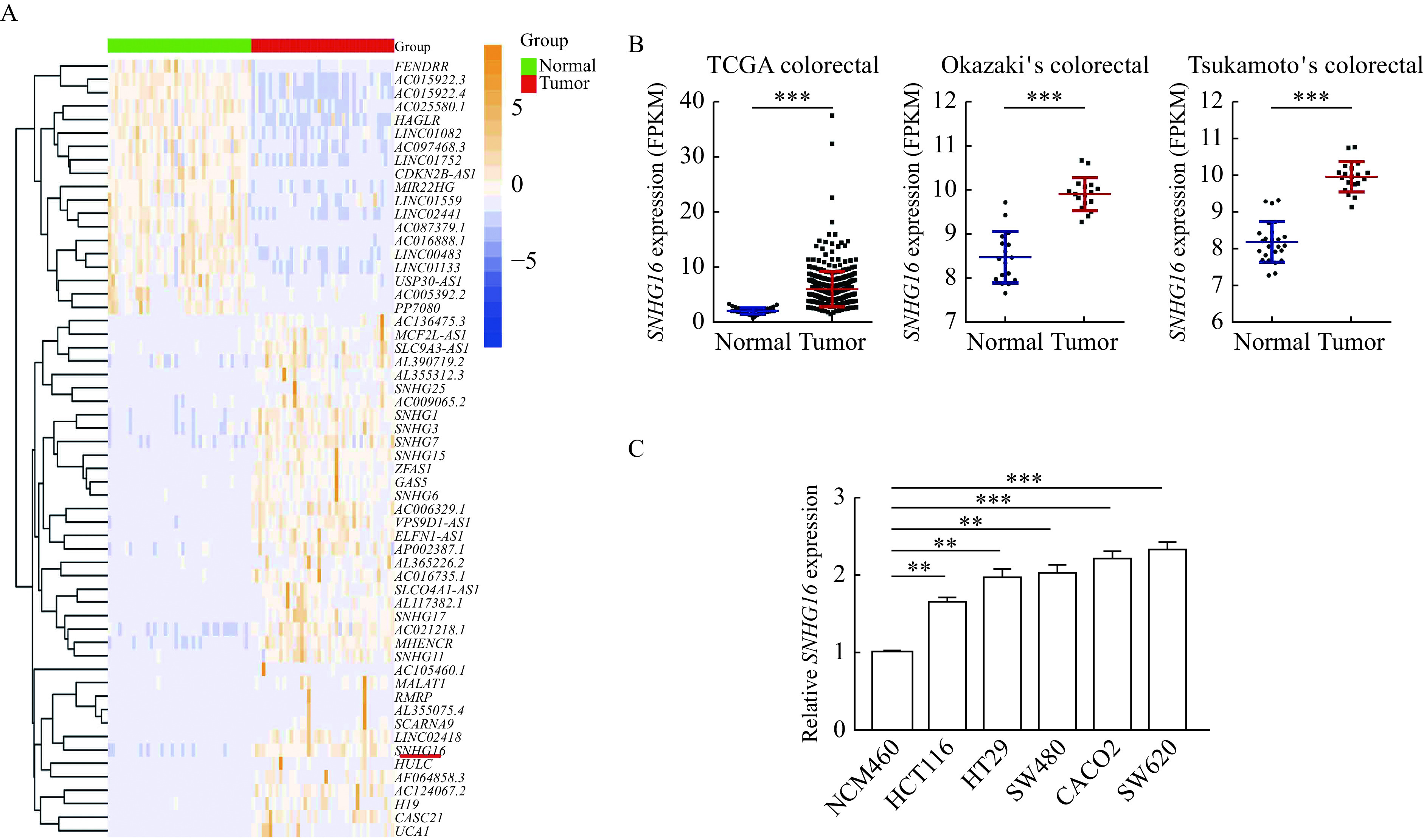
*SNHG16* expression was upregulated in colorectal cancer.

To investigate the differential expression of lncRNAs in CRC, we studied the transcriptome data of human CRC tissue and paracancerous tissue in TCGA. Many abnormally expressed lncRNAs were identified (***Fig. 1A***). Among them, *SNHG16* was selected as the research target due to its relatively high abundance (*P*<0.001,***Fig. 1B***). Then, we analyzed microarray datasets and found that *SNHG16* was also upregulated in two other independent CRC cohorts (*P*<0.001,***Fig. 1B***). Besides, we found that *SNHG16* expression level was associated with the size of the tumor and lymph node metastasis in TCGA CRC cohort (***Supplementary Table 2***, available online). Moreover, we analyzed *SNHG16* expression in CRC cell lines. *SNHG16* expression was upregulated in all five CRC cell lines (SW620, HCT116, HT29, CACO2 and SW480) compared with the human colorectal epithelial cell line NCM460 (*P*<0.01,***Fig. 1C***).

### *SNHG16* promoted CRC cell growth *in vitro*

**Figure 2:**
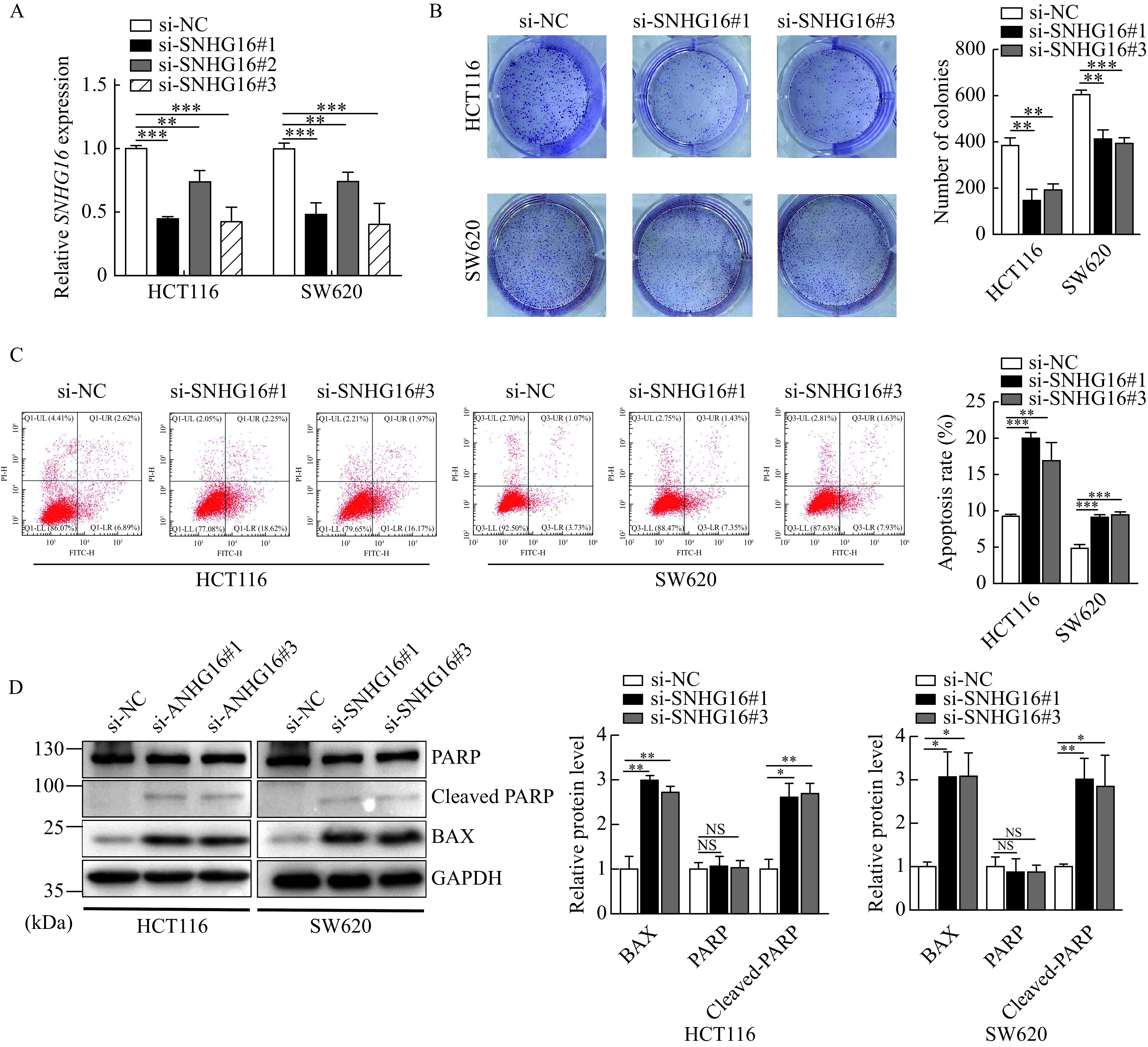
*SNHG16* promoted CRC cell growth *in vitro*.

To determine the potential function of *SNHG16* in CRC, we first knocked down *SNHG16* expression in SW620 and HCT116 cells and verified its efficiency by qRT-PCR (*P*<0.01, ***Fig. 2A***). Colony formation assay results suggested that compared with the control group, *SNHG16* knockdown significantly inhibited SW620 and HCT116 cell growth (*P*<0.01,***Fig. 2B***). We then performed flow cytometry assays to analyze the apoptosis rate. The proportion of apoptotic cells in the *SNHG16* siRNA group was significantly increased compared with the NC group (*P*<0.01,***Fig. 2C***). Western blotting results revealed that the expression levels of the apoptosis-related proteins cleaved PARP and Bax were increased in *SNHG16*-silenced cells. (*P*<0.05,***Fig. 2D***). In addition, transwell and wound healing assays revealed that the migration and invasion abilities of HCT116 cells were significantly reduced when *SNHG16* expression was decreased (*P*<0.01, ***Supplementary Fig. 1A*** and ***B***, available online).

### *SNHG16* promoted CRC cell growth*in vivo*

**Figure 3:**
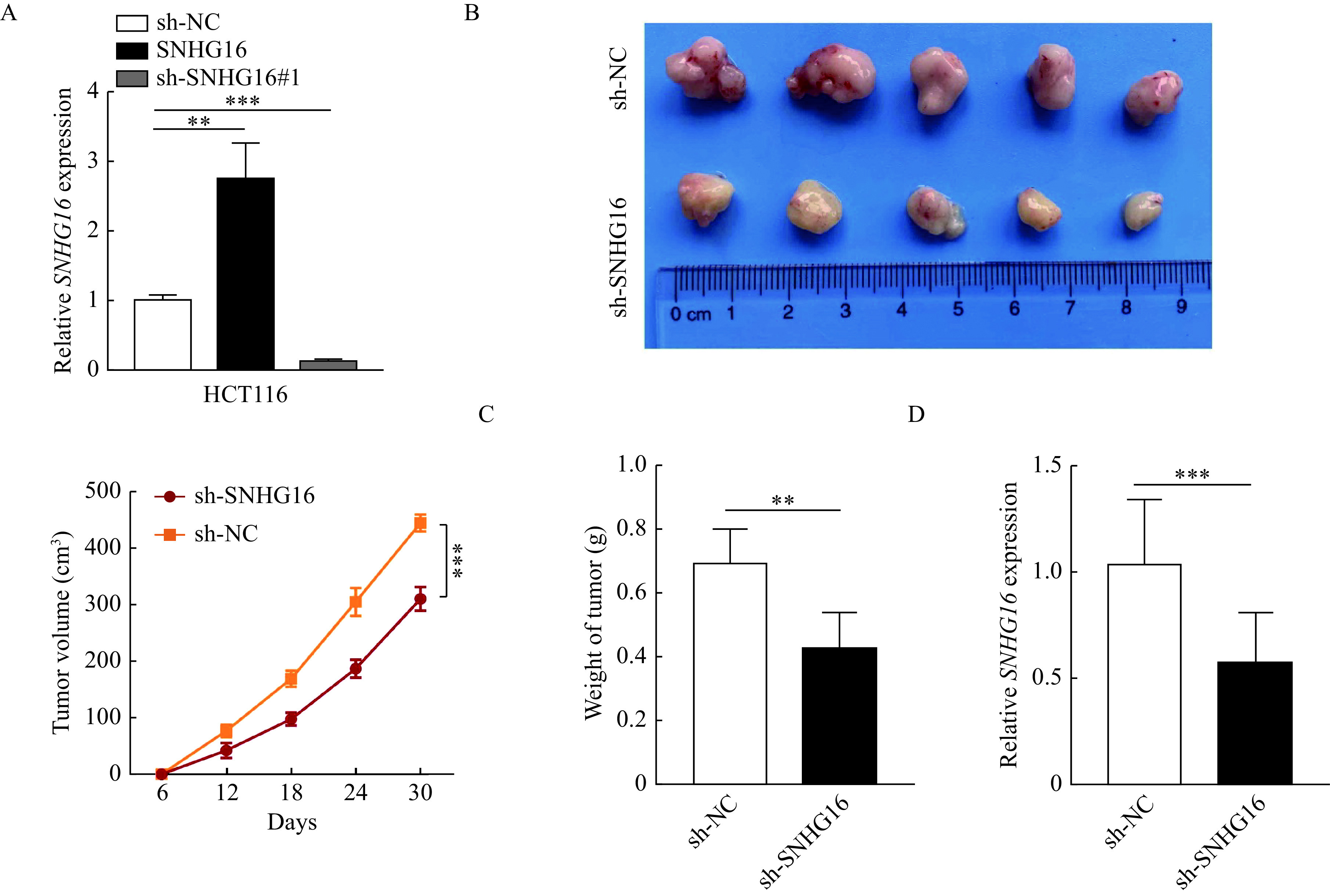
*SNHG16* promoted CRC cell growth *in vivo*.

To further confirm the tumor-promoting effect of *SNHG16* *in vivo*, we established lentiviral-mediated stable in HCT116 cells. As shown in ***Fig. 3A***, the shRNA could silence its expression and *SNHG16* overexpression vectors could increase its expression (*P*<0.01). Then we established animal models by injecting the same amount of HCT116 cells into BALB/c nude mice subcutaneously. At the end of the experiment, the mice were sacrificed. As shown in***Fig. 3B***, intratumorally injection of sh-SNHG16 significantly reduced the size of the tumors (*P*<0.001). In addition, the tumor weight in the sh-SNHG16 group was significantly lower than that in the sh-NC group (*P*<0.01, ***Fig. 3C***). The expression of *SNHG16* was lower in tumors formed by HCT116 cells transfected with sh-SNHG16 vectors (*P*<0.001,***Fig. 3D***).

### *SNHG16* enhanced ABCB1 expression in CRC

**Figure 4:**
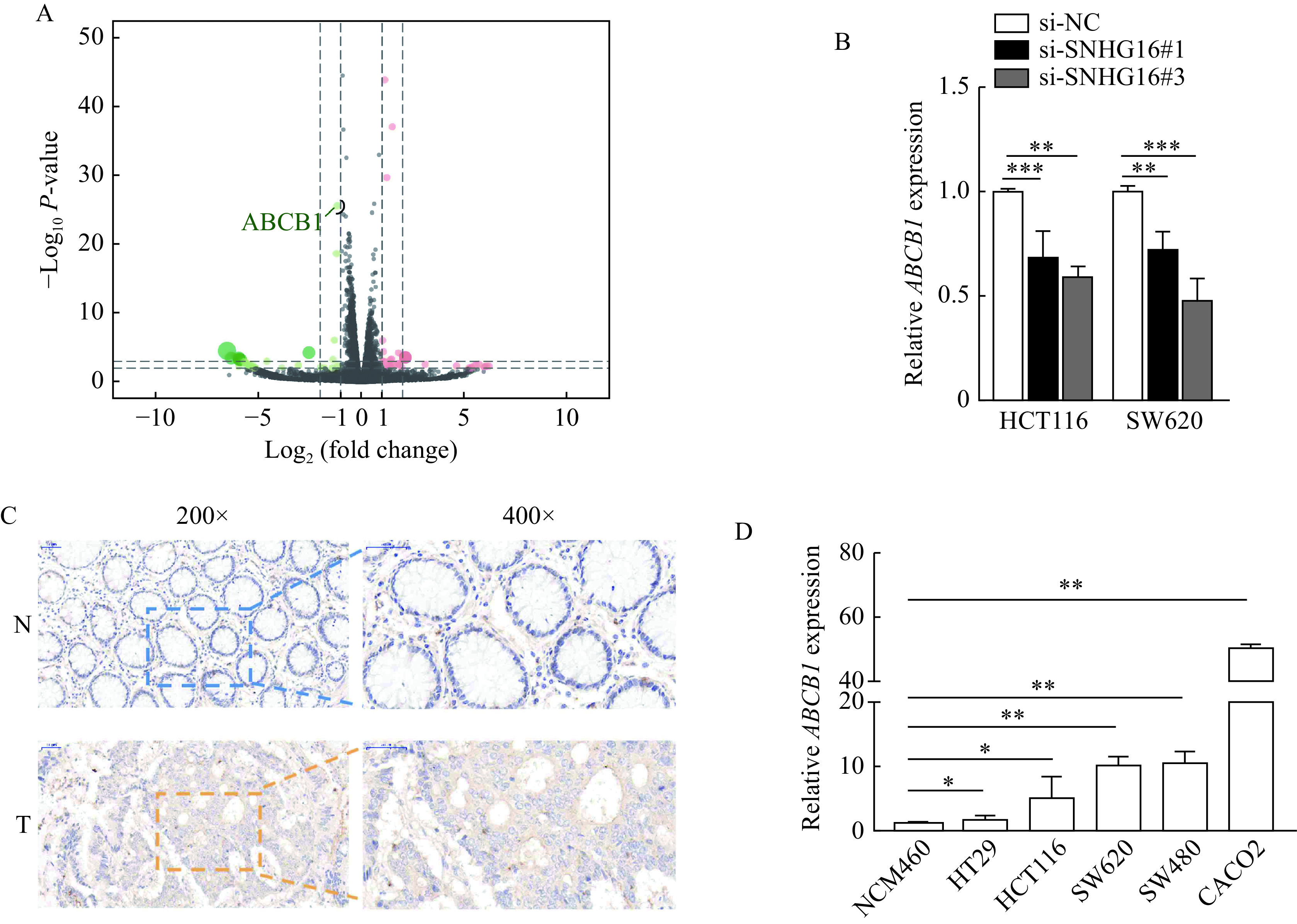
*SNHG16* enhanced the expression of ABCB1.

To identify downstream targets regulated by *SNHG16*, we carried out RNA sequencing analysis of three pairs of HCT116 cells stably transfected with sh-SNHG16 and sh-NC. We then selected ABCB1 for further study, because ABCB1 is a well-known tumor-promoting gene reported by many studies^[16–17]^, and the difference of ABCB1 in sequencing results after *SNHG16* knockdown was statistically significant (***Fig. 4A***). After silencing *SNHG16* in CRC cells, the expression level of ABCB1 was markedly decreased, consistent with the RNA-sequencing results (*P*<0.01,***Fig. 4B***). IHC assays showed that ABCB1 protein levels in CRC tissues were higher than those in adjacent normal tissues (***Fig. 4C***).qRT-PCR results revealed that ABCB1 expression was upregulated in CRC cell lines (*P<*0.05, ***Fig. 4D***).

### *SNHG16* acted as a sponge for miR-214-3p in the cytoplasm

**Figure 5:**
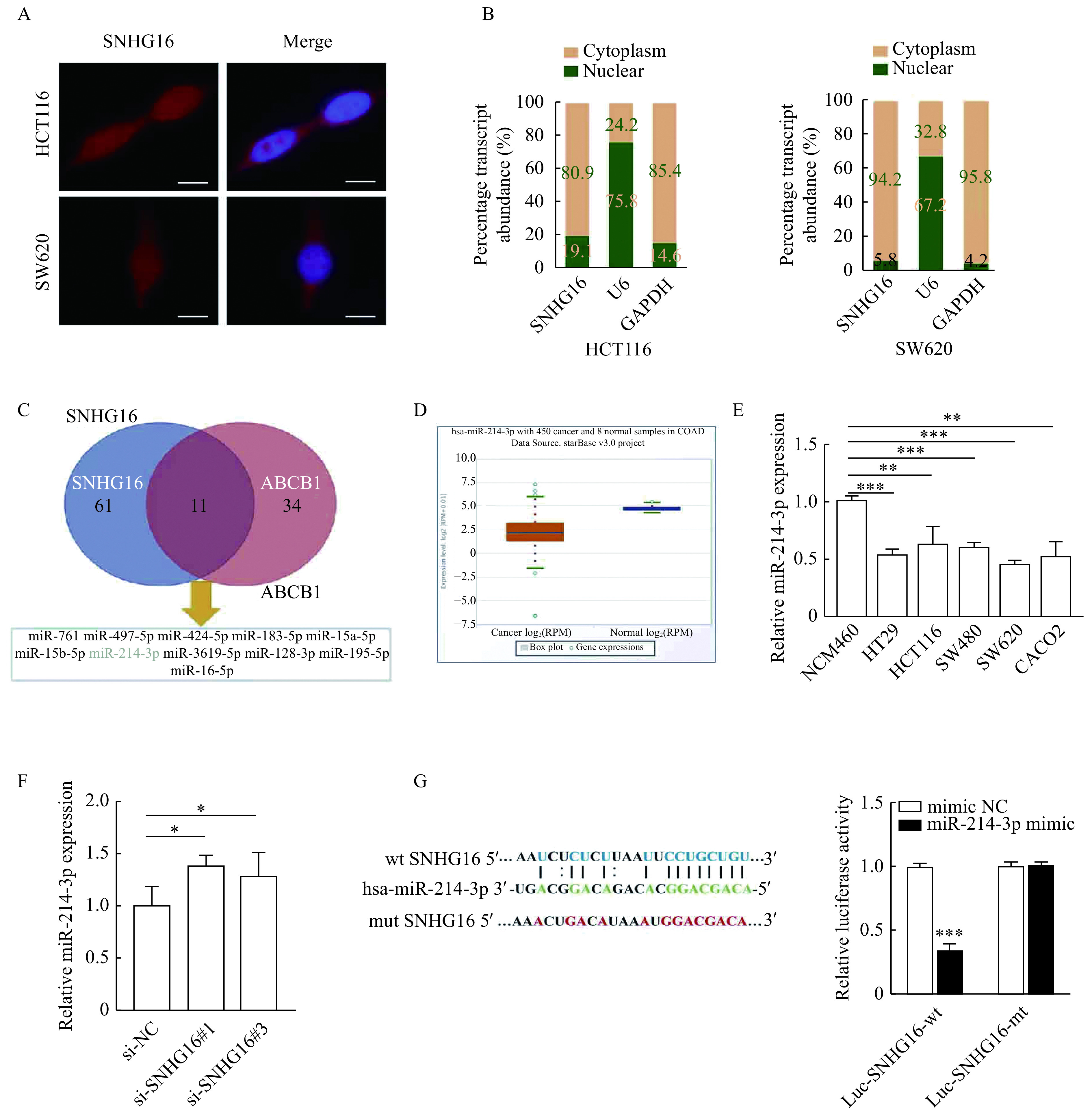
*SNHG16* acted as a sponge for miR-214-3p in the cytoplasm.

To investigate the molecular mechanism by which *SNHG16* promotes the malignant progression of CRC, we analyzed the subcellular localization of *SNHG16* in HCT116 and SW620 cells. We observed that *SNHG16* was mainly expressed in the cytoplasm by FISH and subcellular fractionation assays (***Fig. 5A*** and ***B***). Then we used the ENCORI tool^[18]^ to predict microRNAs that potentially bind to the 3'UTR of *SNHG16* and ABCB1. As shown in ***Fig. 5C***, there were 11 microRNAs that could bind both *SNHG16* and ABCB1, among which miR-214-3p (normal mean=26.69 reads per million mapped reads [RPM], tumor mean=7.29 RPM, *P*<0.001) was markedly reduced in CRC according to the TCGA data (***Fig. 5D***). We analyzed miR-214-3p expression in CRC cell lines by qRT-PCR, and the results were in accordance with the results of the TCGA data (*P*<0.01,***Fig. 5E***). In addition, we found that knockdown of *SNHG16* could significantly increase miR-214-3p expression in HCT116 cells (*P*<0.05,***Fig. 5F***). After that, we conducted a dual-luciferase reporter assay to explore whether miR-214-3p could interact with *SNHG16* directly. The dual-luciferase reporter assay indicated that co-transfection of the wild-type *SNHG16* luciferase vector (Luc-SNHG16-wt) with the miR-214-3p mimics, but not the mutant *SNHG16* vector (Luc-SNHG16-mt), significantly decreased the luciferase activity (*P*<0.001, ***Fig. 5G***).

### Tumor-promoting functions of *SNHG16* were dependent on miR-214-3p/ABCB1

**Figure 6:**
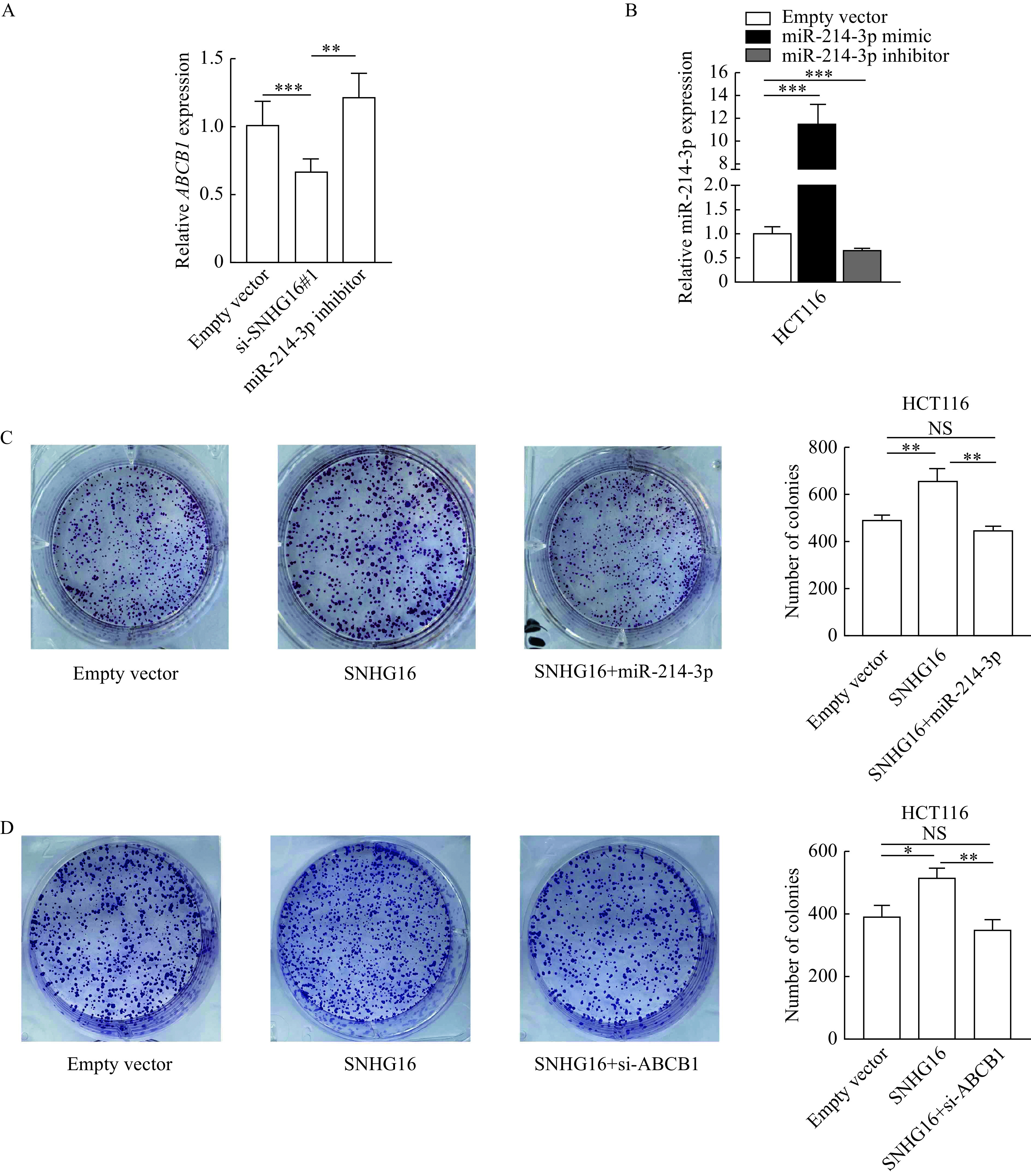
Tumor-promoting functions of *SNHG16* were dependent on miR-214-3p/ABCB1.

To further explore whether *SNHG16* functions as an oncogene by regulating miR-214-3p/ABCB1, we performed qRT-PCR and the results showed that *SNHG16* silencing led to a decrease in ABCB1 expression levels. In addition, co-transfection with miR-214-3p inhibitor rescued the decrease in ABCB1 expression levels caused by *SNHG16* silencing (*P*<0.01, ***Fig. 6A***). Then we performed rescue assays, the efficiencies of mimics and inhibitors of the miR-214-3p are shown in ***Fig.6B*** (*P*<0.001). The results demonstrated that miR-214-3p upregulation could reverse*SNHG16* overexpression-induced promotion of proliferation of CRC cells (*P*<0.01,***Fig. 6C***). Moreover, we designed one small interfering RNAs (siRNAs) targeting ABCB1 (*P*<0.001,***Supplementary Fig. 2***, available online) and the colony formation assay results showed that silencing ABCB1 abolished the increase in cell growth rates induced by *SNHG16* overexpression (*P*<0.05,***Fig. 6D***).

## Discussion

LncRNAs are a heterogeneous group of RNA transcripts with a length of more than 200 nucleotides that are generally considered to have no protein-coding function. However, accumulating evidence indicates that the dysregulated expression of lncRNAs participates in the tumorigenesis and development of CRC^[19–21]^. For example, Sun *et al* showed that lncRNA *TUG1* increased the resistance of CRC stem cells to oxaliplatin^[21]^. Li *et al* found that lncRNA *AFAP1-AS1* promotes cell growth by regulating miR-195-5p/WISP1 axis in CRC^[22]^. Wang *et al* reported that lncRNA UCA1 could be potential diagnostic biomarkers for CRC^[23]^. Hence, lncRNAs may be promising targets for developing effective diagnostic and therapeutic strategies for CRC. In this study, we identified aberrantly expressed lncRNAs in CRC by analyzing TCGA sequencing data and explored the functions of *SNHG16* in CRC. We demonstrated that *SNHG16* expression was upregulated in CRC tissues and cell lines. Functional studies suggested that knockdown of *SNHG16* inhibited cell proliferation but induced cell apoptosis in CRC. In addition, *in vivo* experiments also indicated *SNHG16* promoted tumor growth. Hence, we conclude that *SNHG16* is a cell carcinogenesis regulator in CRC.

In recent years it is reported that genetic factors and exogenous influences, such as diet, drugs and bacterial toxins, as well as other biological and chemical factors, may increase the risk of CRC development^[24]^. Osswald *et al* showed that the risk of CRC development was significantly associated with genetic variations in ABCB1^[25]^. The carcinogenic effect of ABCB1 has also been demonstrated in animal models of breast cancer and liver cancer^[26]^. In this study, we revealed that *SNHG16* could regulate ABCB1 expression by using RNA sequencing and co-expression analysis. ABCB1 was upregulated in CRC tissues and cell lines.

Studies have shown that the subcellular localization of lncRNAs is closely related to their biological functions. LncRNAs located in the cytoplasm mainly act as ceRNA to competitively bind miRNAs, thus regulating the expression of their downstream target genes^[27]^. Here, we found that *SNHG16*, through functioning as a ceRNA in the cytoplasm, modulates ABCB1 expression by binding to miR-214-3p. Subsequently, rescue assays were conducted, and the results suggested that *SNHG16* promoted cell proliferation in CRC by regulating miR-214-3p/ABCB1 axis.

In conclusion, our research revealed that *SNHG16* was overexpressed in CRC tissues and cell lines. Besides, we found that *SNHG16* expression level was associated with the size of the tumor and lymph node metastasis in TCGA CRC cohort. *SNHG16* knockdown inhibited CRC proliferation and promoted apoptosis *in vitro*and *vivo*. Mechanistically, *SNHG16* has an oncogenic role in regulating CRC cell proliferation *via* the *SNHG16*/miR-214-3p/ABCB1 axis, indicating novel potential biomarkers and therapeutic targets for CRC.
